# Phosphatidylserine-microbubble targeting-activated microglia/macrophage in inflammation combined with ultrasound for breaking through the blood–brain barrier

**DOI:** 10.1186/s12974-018-1368-1

**Published:** 2018-11-30

**Authors:** Ranran Zhao, Jie Jiang, Huiwen Li, Min Chen, Renfa Liu, Sujuan Sun, De Ma, Xiaolong Liang, Shumin Wang

**Affiliations:** 1 Ordos Center Hospital, Ordos, 017000 Inner Mongolia China; 2 Department of Ultrasound, Peking University Third Hospital, Beijing, 100191 China; 3 Department of Biomedical Engineering, College of Engineering, Peking University, Beijing, 10019 China

**Keywords:** Ischemia, Blood–brain barrier, Ultrasound-targeted microbubble destruction, Microbubbles, Phosphatidylserine

## Abstract

**Background and purpose:**

Inflammatory reaction plays a crucial role in cerebral ischemia reperfusion (IR) injury. It has been shown that activated microglia long-term existed in cerebral ischemia and induced second injury. Therefore, we hypothesize that prepared phosphatidylserine (PS)-modified microbubbles (PS-MBs) combined with ultrasound-targeted microbubble destruction (UTMD) can safely open the blood–brain barrier (BBB) and target activated microglia for inflammatory area in the later stage of ischemia reperfusion.

**Methods:**

To verify our hypothesis, rat model of IR was established, then the change of activated microglia/macrophage (M/M) and permeability of BBB at 1, 7, 14, and 21 days could be clearly observed post IR. And the activated M/M still can be observed during the whole experiment.

**Results:**

The Evans blue extravasation of BBB gradually declined from day 1 to day 21. Compared to the control group, microbubbles containing PS were taken up more by activated M/M (approximately twofold) both in vitro and in vivo.

**Conclusions:**

PS-MBs combined with ultrasound (US) exposure could safely open BBB, and the resulting PS nanoparticles (PS-NPs) could further target activated M/M in the neuroinflammation.

**Electronic supplementary material:**

The online version of this article (10.1186/s12974-018-1368-1) contains supplementary material, which is available to authorized users.

## Introduction

The blood–brain barrier (BBB) is a major obstacle that prevents therapeutic drugs or genes from being delivered to the central nervous system. Therefore, it is important to develop methods to enhance the permeability of the BBB. The activation of contrast agent microbubbles (MBs) with ultrasound (US) is emerging as a powerful strategy for overcoming physiological barriers associated with drug and gene delivery [1]. It is generally thought that MB expansion and collapse in an acoustic field facilitates the delivery of intravascularly administered drugs/genes to tissue by permeabilizing cellular membranes and/or the microvasculature with permeabilization responses. Ultrasonic activation of MBs induced its conversion into nanoparticles (NPs) and further resulted in increased drug delivery to a variety of tissues, including tumors [2].

Ischemic stroke represents one of the worldwide leading causes of death, and surviving patients often experience long-lasting disabilities. When blood flow is not rapidly restored and ischemic damage develops, injured neurons release damage-associated molecular pattern proteins, leading to the secretion of pro-inflammatory mediators and generation of reactive oxygen species (ROS) from inflammatory cells. Therefore, the early thrombolytic therapy (within 6 h of the time window) to cerebral infarction is very important [3]. However, neuronal damage is primarily due to not only an acute occlusion of cerebral vessels but also ischemic reperfusion (IR) after thrombolytic therapy, which may induce complex inflammatory reaction and may cause second injury to the neuron [4]. Brain macrophages, one of the inflammatory cells, were observed in brain tissue from patients with focal infarction as early as 1 day. Discrepancies existing toward the provenance of brain macrophages, which may be either blood-borne monocytes or resident microglia, and then further differentiate into macrophages, can be also called activated microglia/macrophage (M/M). Activated M/M may become phagocytic aiming to clear the damage and promote repair [5].

Activated M/M can persist for months after ischemia, suggesting a long-term involvement in inflammatory processes at the site of injury. Studies showed that inhibition of inflammation could reduce subsequent neuronal damage [6]. Exposure of phosphatidylserine (PS), normally sequestered in the plasma membrane, is one of the key steps in the recognition and ingestion of apoptotic cells by activated microglia/macrophage, which is mediated by phosphatidylserine-specific receptors (PSRs) [7]. Targeting-activated M/M can be used as a monitoring and therapeutic strategy of inflammation post cerebral ischemia reperfusion (IR) since its existence and persistence in the inflammatory area. It is very difficult to deliver drug to pass through the blood–brain barrier, especially for the water-soluble anti-inflammatory drugs, leading to very low bioavailability [8]. Therefore, much effort has been made in delivering anti-inflammatory drugs through BBB. Ultrasound-targeted microbubble destruction (UTMD) has been demonstrated to be a minimally invasive method for opening BBB via sonoporation effect, facilitating effective delivery of therapeutic agents, such as chemotherapeutic drugs, proteins, and gene [9].

**Fig. 1:**
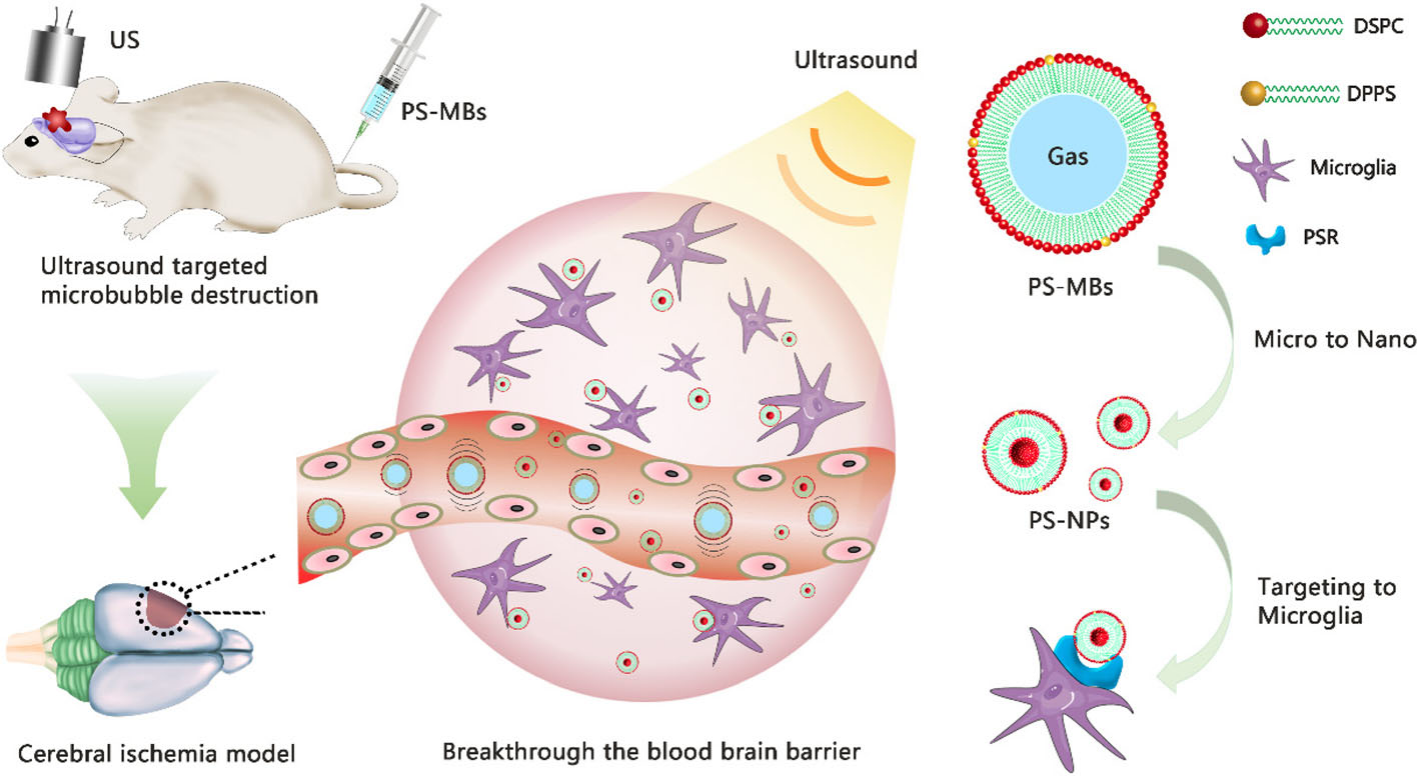
Schematic illustration of phosphatidylserine-microbubble targeting-activated microglia/macrophage in inflammation of stroke. Rats of IR were injected PS-MBs, and transcranial US was performed simultaneously. This process transformed PS-MBs into PS-NPs and acquired ultrasound-targeted microbubble destruction to open BBB, which promoted PS-NPs into the ischemic area

In this study, PS-containing microbubbles (PS-MBs) were first fabricated; safe UTMD was then performed to temporarily open the BBB in the later stage of cerebral infarction. Furthermore, PS-MBs can be converted into PS-NPs upon ultrasound sonication and easily passed through the BBB and then taken up by the activated M/M, achieving novel delivery routes for targeting the inflammation area (Fig. 1). Compared to the conventional phosphatidylcholine-containing liposomes, PS-liposomes used PS as the shell component endowed the resulting MBs with targeting capability, which should be mainly attributed to the PS head group of phospho-l-serine. Our data suggest that, as for peripheral macrophages, PS through its receptor can modulate microglial activation toward an anti-inflammatory phenotype.

## Materials and method

### Preparation of PS-MBs

The phospholipids in powder form (Avanti Polar Lipids Inc., USA) were used in this study without further purification. Briefly, 2-distearoyl-sn-glycero-3-phosphocholine (DSPC), cholesterol, 1,2-distearoyl-sn-glycero-3-phosphoethanolamine-*N*-[methoxy(polyethylene glycol)-2000] (DSPE-PEG2000), and 1, 2-dipalmitoyl-sn-glycero-3-phospho-L-serine (DPPS) were dissolved in ethanol and mixed together to form a lipid mixture at a molar ratio of 50%:40%:5%:5%. Then, the mixture was injected into ultrapure water (1 ml), followed by bath sonication at 60 °C for 5 min, then the solution was dialyzed against PBS with a membrane of 8000–14,000 Da cut-off to completely remove the organic solvent, obtaining the aqueous dispersion of phosphatidylserine nanoparticles (PS-NPs). To prepare PS-MBs, glycerol and 1, 2-propyleneglycol were mixed with the above PS-NP dispersion at a volume ratio of 10%:10%:80% in a 3-ml glass vial. Finally, the vial was filled with perfluoropropane, followed by mechanical agitation for 45 s using a VialMix shaker. PS-MBs were then transferred into a centrifuge tube and separated by centrifugation for removing the residual nanoparticles at 800 r min^−1^ for 10 min and washing with the preparing for several times.

### Characterization of PS-MBs

Fluorescence images were taken by a confocal laser scanning microscopy (Leica TCS SP8 STED 3X, GER). The size distribution and concentration of PS-MBs were measured by a Coulter counter (Multisizer 3 Coulter Counter, Beckman Coulter, Inc., USA). Dynamic light scattering (DLS) measurements were performed by a 90Plus/BI-MAS instrument (Brookhaven Instruments Co., USA). The transmission electron microscope (TEM) sample was prepared by immersing a formvar-coated copper grid into the PS-NP suspension and observed using a FEI TECNAI G2 20 high-resolution transmission electron microscope operating at 200 kV. For in vitro ultrasound imaging, 20 μl PS-MB solution was injected into a latex tube containing 1 ml PBS, and harmonic images were captured by a clinical ultrasound system (DC8, Mindray Medical International Co., Ltd. China).

### In vitro experiment of PS-MBs

Human umbilical vein endothelial cells (HUVECs) were used as a representative of the normal cells, and RAW264.7 cells and macrophage cells as a representative of activated M/M.

#### Cell viability assessment

The cell viability was evaluated by CCK-8 assay (Keygen Biotechnology, China). HUVECs and RAW264.7 cells were plated in the 96-well plates (1.0 × 10^6^ cell per well) and incubated for 24 h before experiments. The medium was replaced with different concentrations of PS-MBs (0, 1 × 10^2^, 1 × 10^4^, 1 × 10^8^, 1 × 10^10^, 1 × 10^12^/ml), and US exposure was performed (1.03 MHz, 50% duty, 1 W/cm^2^, 1 min) and cultured for 4 h. Then, the solution was replaced by fresh medium and cultured for 24 h or 48 h. After treatments, 10 μl of CCK-8 solution was added to each well and cells were incubated for a further 3 h at 37 °C. Absorbance was measured at 450 nm with a microplate reader (Synergy HT, BioTek). The mean optical density (OD) of four wells in each group was used to calculate cell viability as follows: Cell viability (%) = (OD_treatment group_/OD_control group_) × 100. The experiment was performed in triplicate.

#### Cellular uptake of PS-MBs

Murine resident peritoneal macrophages were obtained by peritoneal lavage with 10 ml of RPMI 1640 containing 10% FBS. Cells were incubated 2–3 h and then washed with PBS to eliminate nonadherent cells. Murine resident peritoneal macrophages and RAW264.7 cells were seeded in a 24-well cell culture plate at a density of 5 × 10^4^ cells/well. The cells were incubated with the Cou6-labeled PS-MBs at a concentration of 1 × 10^8^/ml for 4 h with US exposure (1.03 MHz, 50% duty, 1 W/cm^2^, 1 min). The intracellular uptake of the PS-NPs-Cou6 was determined using fluorescence microscope (Leica DMI3000B), and the quantitative analysis was determined using software of Image J (1.8.0_152).

### Establishment of cerebral ischemia reperfusion model

#### Focal cerebral ischemia reperfusion procedure

The male Sprague-Dawley (SD) rats weighing 280 ± 20 g were supplied by Beijing Vital River Laboratory Animal Technology Co., Ltd. Focal cerebral ischemia was conducted by intraluminal middle cerebral artery blockage with a nylon suture, as previously described by Longa et al. and with minor modification by Kawamura et al. [10, 11]. Briefly, the rats were first anesthetized with 2% isoflurane. Rats were placed in the supine position on a heated operating table with body temperature maintained at around 37 ± 0.5 °C. The bifurcation of the left common carotid artery was exposed. The left middle cerebral artery was occluded for 20 min by insertion of a 3.5-mm monofilament suture (Guangzhou Jialing Biological Technology Co., Ltd.) through the internal carotid artery from the external carotid artery. The suture was withdrawn allowing reperfusion.

#### Neurological score

Neurological deficit was scored in each mouse 24 h after ischemic insult in a blinded fashion by two investigators according to the following graded scoring system: 0 = no deficit; 1 = forelimb weakness and torso turning to the ipsilateral side when held by the tail; 2 = IR clings to the affected side; 3 = unable to bear weight on the affected side; and 4 = no spontaneous locomotor activity or barrel rolling.

#### Magnetic resonance imaging and TTC staining of IR rat

Magnetic resonance imaging (MRI) of the rat was then performed. Twenty-four hours after IR, the rat was placed in a dedicated holder and positioned in the isocenter of a 1.5-T MRI scanner (Aspect Imaging, M3TM). During MRI, the rats were anesthetized with 2% isoflurane by mechanical ventilation. Respiration and heart rate were monitored during MRI measurements, and body temperature was maintained at 37.0 ± 0.5 °C. T2-weighted images were acquired with a fast spin echo sequence (TR = 3000 ms, TE = 154 ms, matrix size = 256 × 256, field of view = 6 cm × 6 cm, slice thickness = 2 mm, NEX = 4).

TTC staining was performed 24 h after IR to determine the infarction volume. Brain tissues were cut into 2-mm-thick coronal sections and immersed in 2% solution of TTC (Sigma, St. Louis, MO, USA) for 30 min at 60 °C. Then, the stained slices were fixed by immersion in 4% formaldehyde solution. The infarct area of each section was photographed.

### Evaluation of changes in activated M/M and BBB after IR

#### Tracking the activated M/M in cerebral infarction

SD male rats post IR were equally and randomly assigned to the following five groups: sham, 1, 7, 14, and 21 days. Rats were anesthetized by intraperitoneal injection of sodium pentobarbital (100 mg/kg); the animal was perfused with normal saline followed by 4% paraformaldehyde in 0.1 M PBS, pH 7.2–7.4, for 20 min. Then, the frozen brain tissue was cut into serial coronal sections and incubated with anti-Iba1 fluorescent antibody. Sections were then stained with 4,6-diamidino-2-phenylindole (DAPI). The samples were investigated with a fluorescence microscope. The numbers of Iba1-positive cells per field were manually counted and averaged.

#### Assessment of the BBB after IR

At different time points after IR (Sham, 1, 7, 14, and 21 days), rats were injected via the tail vein with 5 ml/kg of 2% Evans blue (EB). After 2 h of IRculation, the animal was anesthetized by intraperitoneal injection of sodium pentobarbital (100 mg/kg) and perfused with normal saline followed by 4% paraformaldehyde in 0.1 M PBS, pH 7.2–7.4, for 20 min. Then, the brain tissue was cut into 2-μm coronal sections, photographed and homogenized in 1 ml of 50% trichloroacetic acid (wt/vol). After centrifugation (12,000×*g*, 20 min), supernatant was collected and mixed with ethanol (1:3, *V*/*V*). The concentration of Evans blue was determined by measuring the 610-nm absorbance, and tissue content of Evans blue was quantified from a linear standard curve and expressed in terms of Evans blue (ng)/tissue (g).

### Application of transcranial UTMD of PS-MBs for cerebral IR

#### Assessment of PS-MBs for opening BBB

Rats of IR were sedated with a 2% isoflurane nose cone and divided into five different groups (0, 20, 50, 100, 200 μl/mouse of PS-MBs). Hair was removed by depilatory lotion in rats. Detaining needle was placed in the tail vein to deliver PS-MBs, and transcranial US was carried out simultaneously with US, which was generated by a therapeutic US system (KTAC-4000, WELLD, NEPA GENE, Japan) (1.03 MHz, 50% duty, 3 W/cm^2^, 60 s). Then, 2% EB solution was added via detaining needle. After 2 h, the brain tissues were collected and treated as mentioned above.

#### Target enrichment ability of PS-MBs in cerebral infarction

Rats of IR were sedated with a 2% isoflurane nose cone and divided into six different groups, including control, US, MBs, PS-MBs, US+MBs, and US+PS-MBs (*n* = 5). The microbubbles labeled with DiR were injected in the tail vein (200 μl/mouse). After 24 h, the brain tissues were cut into 2-μm sections. Fluorescence images of the brain sections were acquired and analyzed using an IVIS Imaging Spectrum System (PerkinElmer, U.S.) under certain parameters (*λ*ex = 748 nm, *λ*em = 780 nm). Then, the frozen brain tissue was incubated with anti-Iba1 fluorescent antibody. Sections were then stained with 4,6-diamidino-2-phenylindole (DAPI). The samples were investigated with a fluorescence microscope.

#### Statistical analysis

Statistical analysis was performed by two-tailed Student’s *t* test for two groups, and one-way analysis of variance for multiple groups. A value of *P* < 0.05 was considered statistically significant.

## Results

### Preparation and characterization of PS-MBs

**Fig. 2:**
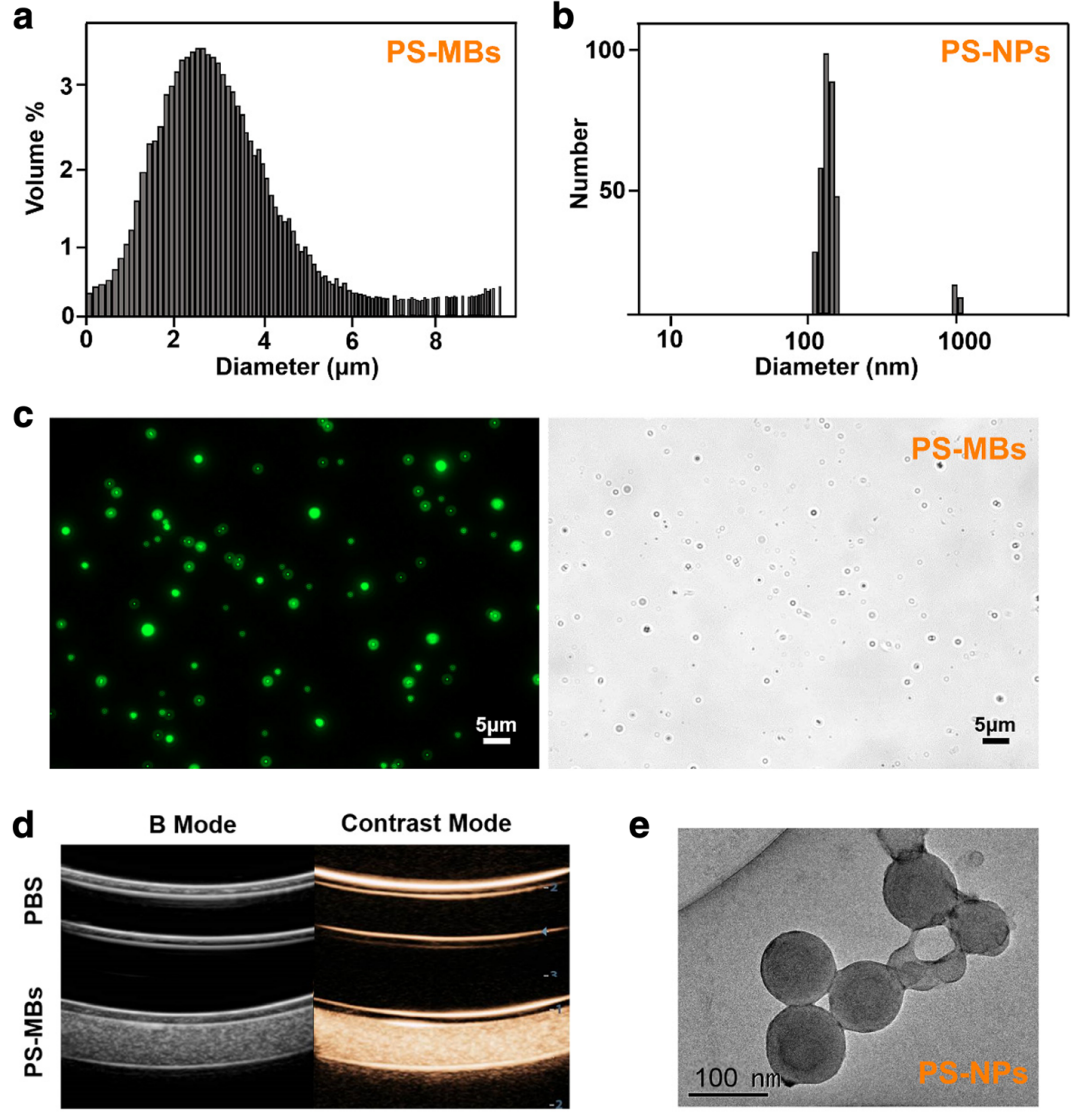
Characterizations of PS-MBs before and after LFUS exposure. Size distributions of **a** PS-MBs and **b** PS-NPs. **c** Fluorescence images of PS-MB-labeled Cou6. **d** Contrast-enhanced ultrasound images of PBS and PS-MBs. **e** The transmission electron microscope images of PS-MBs after US sonication (PS-NPs)

PS-MBs were fabricated from the mixture of DSPC, cholesterol, DSPE-PEG200, and DPPS through an ethanol injection method, followed by filling containers with perfluoropropane (C_3_F_8_). The PS-MBs showed spherical morphology with a hydrodynamic diameter of 4.3 ± 1.1 μm and a positive surface charge of − 22 ± 1.2 mV (Fig. 2a, c). PS-MBs can greatly enhance the US contrast signal, exhibiting good ultrasound contrast effect (Fig. 2d). To further study the shape and size changes of PS-MBs upon UTMD process, low-frequency pulsed ultrasound (LFUS) was used to sonicate the samples, resulting in the complete destruction of PS-MBs; the size distribution of PS-MBs after LFUS exposure was tested by a dynamic light scattering (DLS) measurement, showing mean diameter of about 100 nm (Fig. 2b, e), suggesting the successful conversion of PS-MBs to PS-nanoparticles (PS-NPs) upon ultrasound sonication, which play an important role for PS-NPs that selectively accumulate at the tumor site in vivo.

### Biocompatibility and cell targeting of PS-MBs in vitro

**Fig. 3:**
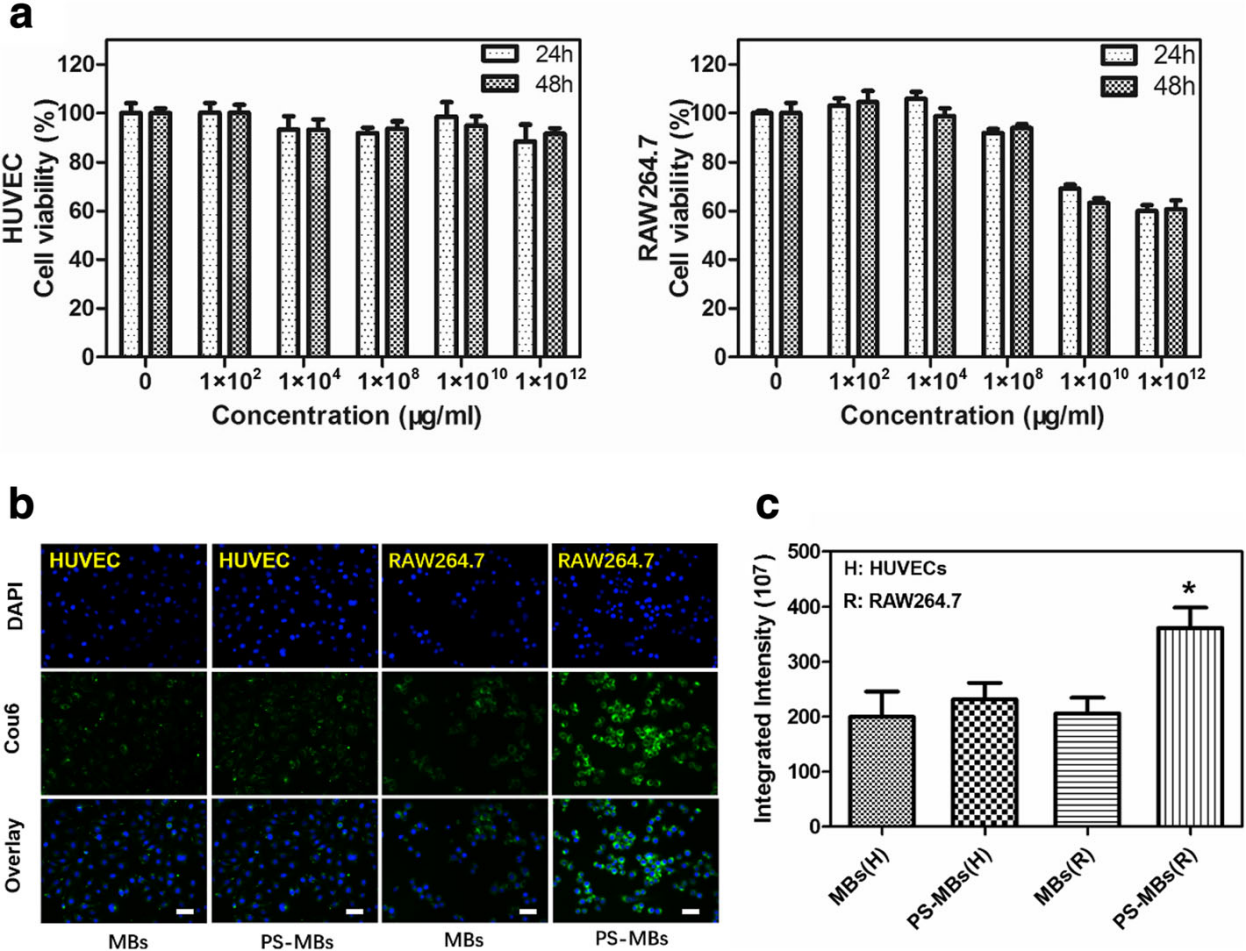
Biocompatibility and intracellular distribution of PS-MBs. **a** Cell viability of HUVEC and RAW264.7 cells under different concentration of PS-MBs using CCK-8 assay. **b** Fluorescence examination of the intracellular distribution of MBs with or without PS-labeled Cou6 at a concentration of 1 × 10^8^/ml (scale bar 30 μm). **c** Quantitative analysis of fluorescence intensity of internalization MB-labeled Cou6 with or without PS in RAW264.7 cells by Image J. (*P < 0.05 versus control)

The cytotoxicity of PS-MBs to HUVECs and RAW264.7 cells was first investigated (Fig. 3a). At concentration below 1 × 10^8^/ml, PS-MBs showed negligible cytotoxic effect to the proliferation of both HUVECs, and RAW264.7 even prolonged the incubation time to 48 h, showing good biocompatibility. However, the cell viability of RAW264.7 was greatly decreased to nearly 70% when the sample concentration was above 1 × 10^10^/ml.

To investigate the targeting capability of PS-MBs to RAW264.7 cells, cou6-labeled PS-MBs were both cultured with HUVECs and RAW264.7 cells; conventional MBs were used as the control. After incubation for 4 h, the intracellular uptake behavior of PS-MBs was observed and quantified by fluorescence microscope (Fig. 3b, c). Little uptake of MBs and PS-MBs could be observed for the normal HUVEC cells, without significant difference. In stark contrast, fluorescent PS-MBs were clearly observed inside the cells as green spots distributed in the cytoplasm. After the nuclei of the tumor cells were stained with 4′,6-diamidino-2-phenylindole (DAPI; blue in Fig. 3), PS-MBs were found to be distributed throughout the entire cytoplasm, while there was only a little PS-MB uptake for the conventional MBs; the average fluorescence intensity of the PS-MB group was two times that of the MB group, exhibiting excellent RAW264.7 cell targeting capability of PS-MBs.

### Evaluation of activation of M/M and blood–brain barrier after focal cerebral ischemia reperfusion

**Fig. 4:**
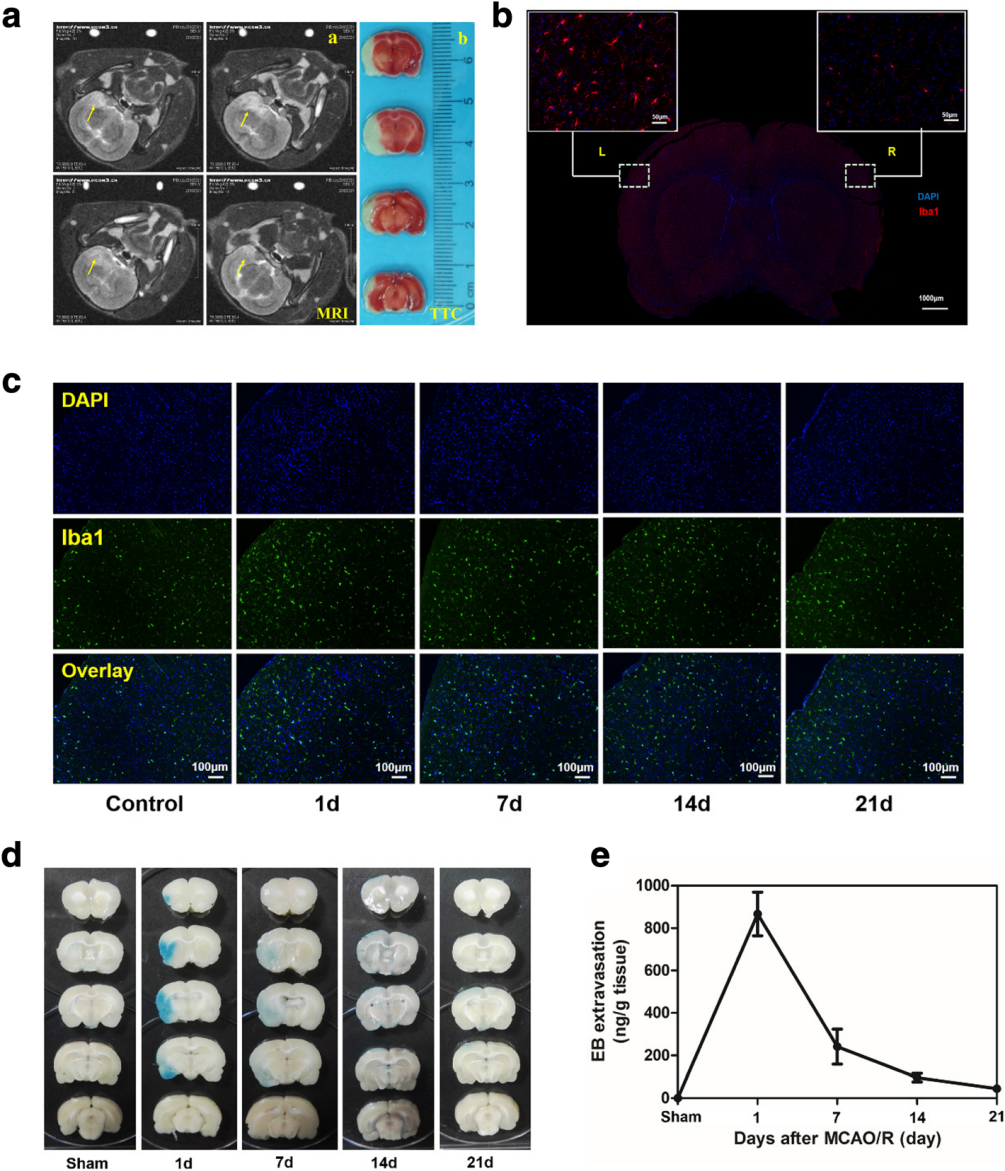
Evaluation of activation MM and blood brain barrier afterfocal cerebral ischemia reperfusion: **a** Evaluation of cerebral ischemia rat model by MRI-T2 (Four scanning imaging), as arrow (**a**) and 2,3-5-Triphenyltetrazolium chloride (TTC) for cerebral infarction (**b**). **b** Activation MM change 24h after focal cerebral ischemia reperfusion. Immunofluorescence staining for Iba1(red) with DAPI (blue). Left brain: cerebral infarction site. Right brain: control. **c** Activation MM change at 1, 7, 14, 21 days after focal cerebral ischemia reperfusion. Immunofluorescence staining for Iba1 (green) with DAPI (blue). EB extravasation in the brain as a function of BBB 24h after focal cerebral ischemia reperfusion. **d** The rat were injected into the tail vein with 5 ml/kg of 2% EB: Distribution of BBB disruption by extravasation of EB and (**e**) the amount of EB extravasation

The rat of cerebral ischemia-reperfusion model was successfully established and confirmed by MRI and TTC staining (Fig. 4a). Compared to the normal hemisphere (right), Iba1-labeled activated M/M in the left cerebral infarction could be observed with number increase and shape change (from IRcular to dendritic) (Fig. 4b). The track of activation M/M was further studied at 1, 7, 14, and 21 days post IR. Activated M/M persisted in the IR area from the first day to the 21st day (Fig. 4c and Additional file 1: Figure II A). On the other hand, EB extravasation was usually used to measure the BBB function of the brain. BBB was opened within 24 h after cerebral ischemia, and the permeability gradually decreased with time increase to 21 days (Fig. 4d, e).

### Application of transcranial UTMD of PS-MBs for cerebral IR

**Fig. 5:**
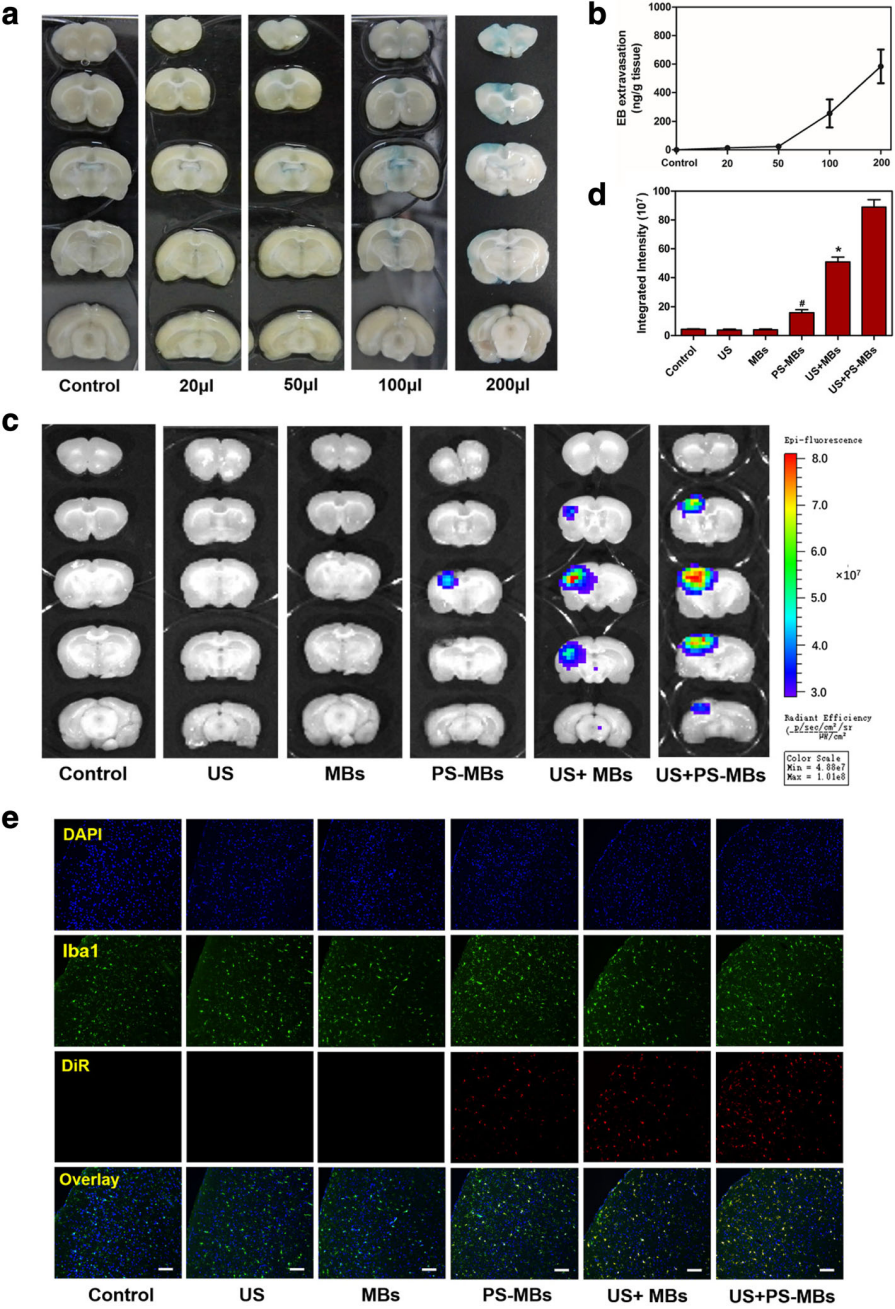
The experiment of microbubbles in vivo through ultrasound. EB extravasation in the brain as a function of BBB after US exposure (1.0 MHz, 3 W/cm^2^, 60 s). **a** Distribution of BBB disruption by extravasation of EB. Left brain: US-exposed site. **b** The amount of EB extravasation. **c** Fluorescence imaging in vivo at different time points after intravenous administration of PS-MBs (200 μl/mouse) with or without US exposure. **d** Quantitative analysis of fluorescence intensity for cerebral infarction site of different groups. **e** Immunofluorescence staining for activation M/M and fluorescence intensity of MB-labeled DiR for cerebral infarction site in different groups (*P < 0.05 versus control)

UTMD technology has unique advantages such as image-guided delivery and enhanced vascular and cellular permeability. In particular, UTMD induces transient and reversible opening of the blood–brain barrier (BBB), facilitating drug release from microbubbles and extravasation into brain tissue [12]. To evaluate BBB permeability after TUMD, after 21 days, rats of IR were injected different concentrations of PS-MBs and transcranial US was performed simultaneously. The result of EB extravasation showed that as the concentration increased, the permeability of BBB also increased (Fig. 5a, b). NIR fluorescence imaging was then performed to trace the biodistribution of PS-MBs post US sonication. Ultrasound exposure could significantly improve the local BBB permeability, and significantly, enrichment in cerebral infarction with subsequent cycle could be observed in the PS-MB group (Fig. 5c, d). Immunohistochemical staining showed a similar result; the PS-MBs + US group was observed with significant fluorescence intensity, which further confirms that PS-MBs could better target activated M/M in IR rats (Fig. 5e and Additional file 1: Figure II B).

## Discussion

In this study, the process of blood–brain barrier opening and microglia activation at different times after IR was described. The inflammatory reaction occurred immediately after IR, and the activated M/M reacted quickly as early as 1 day, which lasted during the whole observation period. This meant that inflammation may further damage neurons in the later period of IR. In addition, in our research, we found that BBB after IR was gradually closed from the early stage to the late stage. Obviously, the integrity of BBB greatly influenced the delivery of anti-inflammatory drugs or contrast agents.

Inflammatory cells were recruited continuously from resident and IRculatory after cerebral ischemia, including activated M/M, neutrophils, and lymphocyte, which resist the harmful substances produced by ischemia and hypoxia [13]. Iba1 is usually used to identify the cell markers that activated M/M [14]. In this study, the rat brain at 24 h post IR was treated with immunofluorescence-labeled Iba1. It was found that the amount of activated M/M increased significantly in the left cerebral ischemia area, and the shape appeared to be dendritic (Fig. 4b). It has been reported that activated M/M with dendritic morphology is easy to recruit and travel and further shows active inflammatory response in ischemic area immediately after ischemia [15]. To track the change of activated M/M in the long term, rats were sacrificed for its brain needed in immunofluorescence experiments at 1, 7, 14, and 21 days post IR (Fig. 4c and Additional file 1: Figure II A). Studies showed that activated M/M can clean up phagocytosis necrotic and apoptotic cells for neuron repair in the late stage of ischemia [16]. However, inflammatory factors of activated M/M could lead to excessive inflammatory immune cascade and aggravate neuronal damage, which is not conducive to long-term prognosis of IR [17]. Therefore, monitoring inflammatory reactions and inhibiting later-stage inflammatory response are studied by many scholars, in which the methods for targeting activated M/M become the focus of research [18].

In our research, we used characteristics of activated M/M to identify the apoptotic cells by PS externalization. PS containing microbubbles (PS-MBs) was successfully fabricated, with a spherical structure (2–5 μm) filled with C_8_F_8_ gas (Fig. 2a, c), upon US exposure; it can be converted into nanoparticles (~ 100 nm) (Fig. 2b, e). As Fig. 2d shows, PS-MBs can greatly enhance the US contrast signal in vitro, allowing to be used as an excellent ultrasound contrast agent. To study the targeting capabilities of PS-MBs, cell uptake experiment was performed with both HUVEC cells and RAW264.7 cells. After incubation with PS-MBs for 24 h or 48 h, it was observed that viability of HUVECs was all above 90% within concentration of 1 × 10^12^/ml, indicating good biocompatibility (Fig. 3a). In contrast, for RAW246.7, the cell viability obviously decreased to about 70% when the sample concentration was above 1 × 10^10^/ml. As reported, macrophages would be induced to be dead after phagocytosis of apoptotic cells, which includes the autophagy and apoptosis process of macrophages themselves [19]. We speculate that when macrophages phagocytose too many PS-MBs, it will initiate the death process due to exceeding the metabolic load of cells (Fig. 3a). To verify this, the intracellular distribution of Cou6-labeled PS-MBs was studied; MBs without PS were used as control. As shown in Fig. 3b, c and Additional file 1: Figure I, the fluorescence intensity in the PS-MB groups was nearly twofold higher than that in the control group in RAW264.7 and murine resident peritoneal macrophages. It suggested that PS possessed excellent target capability to macrophages in vitro.

The EB extravasation method was used to detect the changes of BBB at different time points after IR. The result showed the integrity of BBB was destroyed after IR (Fig. 4d), well consistent with the research [20]. As Fig. 4d, e shows, EB exudation reduced gradually with time increase (from day 1 to day 21), and there was no BBB exudation observed at day 21, which meant BBB recovery and the balance of the brain was maintained. However, the balance can partly restrict the entry of anti-inflammatory drugs into the brain. Thus, opening the BBB has become a research focus in drug delivery. Microbubbles can shrink and expand under ultrasound. When a certain pressure is reached, they burst and produce high energy, which could mechanically open the tight junctions between endothelial cells [21]. This technology was applied to drug delivery and gene transfection for brain diseases, including brain tumors and Alzheimer’s disease [22]. In this study, PS-MSs was injected into the rat via the tail vein, and the application of ultrasound sonication can increase local permeability of BBB. Moreover, the permeability increased with concentration of PS-MBs (Fig. 5). Research showed that UTMD could temporarily open BBB and close within 6–24 h [23], which meant UTMD is a safe and effective way for drug transportation in the brain. In the in vivo targeting research, we used near-infrared dye DiR to label microbubbles and injected via the tail vein. After ultrasound irradiation, brain tissue of rat was isolated, then fluorescence image was captured. The results showed that compared with MB groups, the fluorescence intensity of the PS-MB groups was obviously improved under the same US condition (Fig. 5c), further confirming the local ultrasound-targeted PS-MB destruction could increase the opening of BBB and promote the delivery of PS-NPs to the inflammation region. Moreover, immunofluorescence of brain tissue proved that the highest amount of activated M/M cells was labeled by PS-carrier with the assistance of ultrasound (Fig. 5e; Additional file 1: Figure II B), showing more effectivity than the conventional MBs.

## Conclusion

The PS-MBs fabricated here can safely open the BBB, showing targeting property of activated M/M both in vitro and in vivo. It has been reported that MBs can be used as effective drug carriers, so this study may provide a foundation for further implementation of targeted drug delivery and inflammatory imaging in the cerebral ischemia area. Our next goal is to study the anti-inflammatory treatments for cerebra ischemia.

## Additional file

Additional file 1: **Figure I.** Fluorescence examination of the intracellular distribution of MBs with or without PS-labeled Cou6 at a concentration of 1 × 10^8^/ml in murine resident peritoneal macrophages (scale bar 10 μm). II. Quantitative analysis of fluorescence intensity of (A) activation MM change at 1, 7, 14, and 21 days after focal cerebral ischemia reperfusion. Immunofluorescence staining for Iba1 (B) activation M/M at cerebral infarction site in different MB groups. (*P < 0.05 versus control). (DOC 3510 kb)

## Abbreviations

BBB: Blood–brain barrier
DAPI: 4,6-Diamidino-2-phenylindole
DLS: Dynamic light scattering
DPPS: 1, 2-Dipalmitoyl-sn-glycero-3-phospho-L-serine
DSPC: 2-Distearoyl-sn-glycero-3-phosphocholine
DSPE-PEG2000: 1,2-Distearoyl-sn-glycero-3-phosphoethanolamine-*N*-[methoxy(polyethylene glycol)-2000]
EB: Evans blue
HUVECs: Human umbilical vein endothelial cells
IR: Cerebral ischemia reperfusion
IR: Ischemia reperfusion
M/M: Microglia/macrophage
MBs: Microbubbles
PS: Phosphatidylserine
PS-MBs: Phosphatidylserine-modified microbubbles
PS-NPs: Phosphatidylserine nanoparticles
PSRs: Phosphatidylserine-specific receptors
ROS: Reactive oxygen species
SD: Sprague-Dawley
US: Ultrasound
UTMD: Ultrasound-targeted microbubble destruction
