# Supplementary material for: Phosphatidylserine-microbubble targeting-activated microglia/macrophage in inflammation combined with ultrasound for breaking through the blood–brain barrier
Source: J Neuroinflammation. 2018 Nov 30;15:334. doi: 10.1186/s12974-018-1368-1 (PMC6271401; doi:10.1186/s12974-018-1368-1)
Supplement: Supplementary file 1 — Figure I. Fluorescence examination of the intracellular distribution of MBs with or without PS-labeled Cou6 at a concentration of 1 × 10^8^/ml in murine resident peritoneal macrophages (scale bar 10 μm). II. Quantitative analysis of fluorescence intensity of (A) activation MM change at 1, 7, 14, and 21 days after focal cerebral ischemia reperfusion. Immunofluorescence staining for Iba1 (B) activation M/M at cerebral infarction site in different MB groups. (*P < 0.05 versus control). (DOC 3510 kb) [file 12974_2018_1368_MOESM1_ESM.doc]

**Supporting Information**

**Phosphatidylserine-microbubble targeting activated microglia/macrophage**

**in inflammatory combined with ultrasound for breaking through the blood brain barrier**

Ranran Zhao^1,2 , #^, Jie Jiang^2,#^, Huiwen Li^1,#^, Min Chen^3^, Renfa Liu^3^, Sujuan Sun^1^, De Ma^1^, Xiaolong Liang^2^,*, Shumin wang^1,2^,*

#These authors contributed equally to this work

^1^Ordos Center Hospital, Ordos, Inner Mongolia 017000, China

^2^Department of Ultrasound, Peking University Third Hospital, Beijing 100191, China

^3^Department of Biomedical Enginering, College of engineering, Peking University, Beijing 10019, China

**Corresponding author:**

Shuminwang, PhD

Department of Ultrasound

Peking University Third Hospital

Beijing 100191, China

Fax: (+86)10-82265851

1. mail: [shuminwang2014@163.com](mailto:shuminwang2014@163.com)

Xiaolong Liang, PhD

Department of Ultrasound

Peking University Third Hospital

Beijing 100191, China

Fax: (+86)10-82265851

mail: [xiaolong_liang@bjmu.edu.cn](mailto:xiaolong_liang@bjmu.edu.cn)

**Individual email addresses of authors**

**Ranran Zhao:** [zrr822@sina.com](mailto:zrr822@sina.com)

**Jie Jiang:** [jiangjie_us@163.com](mailto:jiangjie_us@163.com)

**Huiwen Li:** [18604876566@163.com](../../../../F:%5C课题Stroke%5C文章%5C20180926%5C18604876566@163.com)

**Min Chen:** [chenmin168cool@163.com](mailto:chenmin168cool@163.com)

**Renfa Liu:** [hitlrf@sina.cn](mailto:hitlrf@sina.cn)

**Sujuan Sun:** [327185295@qq.com](mailto:327185295@qq.com)

**De Ma:** [381963957@qq.com](mailto:381963957@qq.com)

**Xiaolong Liang:** [xiaolong_liang@bjmu.edu.cn](../%20xiaolong_liang@bjmu.edu.cn)

**Shumin Wang:** [shuminwang2014@163.com](mailto:shuminwang2014@163.com)


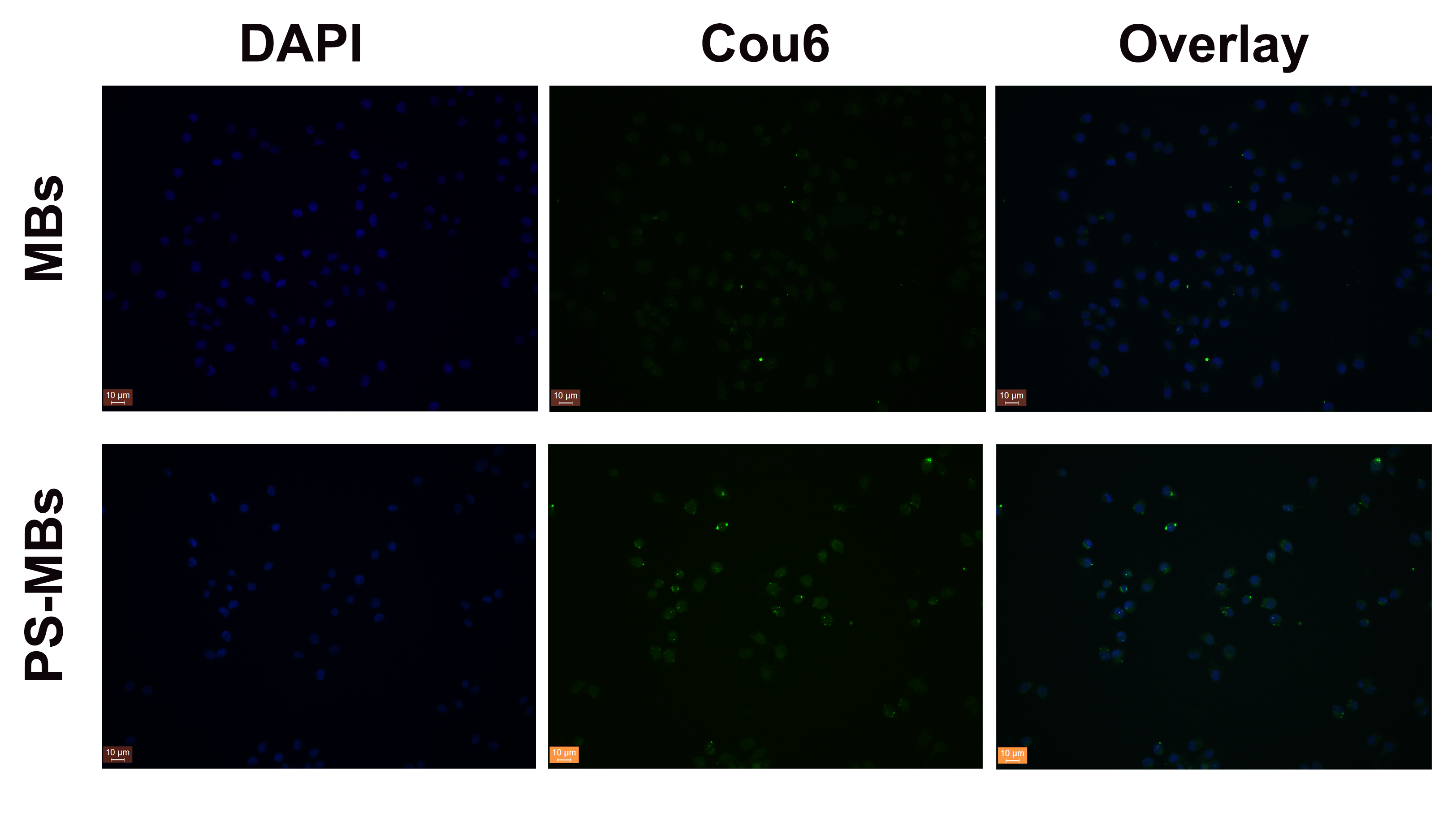


II **I**. (B) Fluorescence examination of the intracellular distribution of MBs with or without PS labeled Cou6 at a concentration of 1×10^8^ /ml in murine resident peritoneal macrophages (scale bar 10 μm).


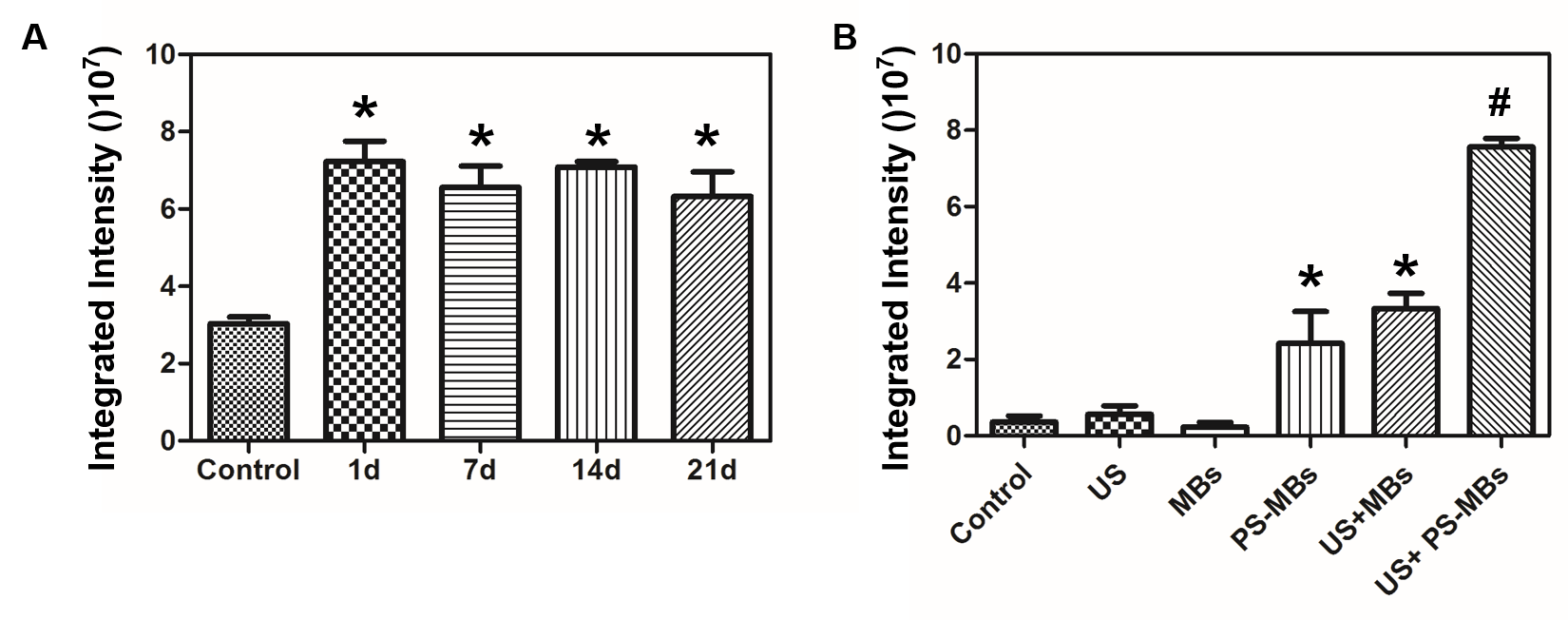


**Figure II**. Quantitative analysis of fluorescence intensity of (A)activation MM change at 1, 7, 14, 21days after focal cerebral ischemia reperfusion. Immunofluorescence staining for Iba1. (B) activation M/M at cerebral infarction site in different MBs groups. (**P* < 0.05 versus Control).
